# Combinatorial Control of Transgene Expression by Hypoxia-Responsive Promoter and MicroRNA Regulation for Neural Stem Cell-Based Cancer Therapy

**DOI:** 10.1155/2014/751397

**Published:** 2014-04-17

**Authors:** Yumei Luo, Detu Zhu

**Affiliations:** Key Laboratory for Major Obstetric Diseases of Guangdong Province, The Third Affiliated Hospital of Guangzhou Medical University, 63 Duobao Road, Liwan District, Guangzhou City, Guangdong Province 510150, China

## Abstract

Owing to their strong migratory capacity, tumor tropism, and tumor inhibitory effect, neural stem cells (NSCs) have recently emerged as one of the most attractive gene delivery vectors for cancer therapy. However, further animal studies found that proportional NSC vectors were distributed to nontarget organs after intravenous injection and the nonspecific transgene expression led to significant cytotoxic effects in these organs. Hence, an expression cassette that controls the transgene expression within NSC vectors in a tumor site-specific manner is desired. Considering hypoxia as a hallmark of tumor microenvironment, we have developed a novel NSC vector platform coupling transcriptional targeting with microRNA (miRNA) regulation for tumor hypoxia targeting. This combinatorial vector employed a hypoxia-responsive promoter and repeated targeting sequences of an miRNA that is enriched in NSCs but downregulated upon hypoxia induction to control the transgene expression. This resulted in significantly improved hypoxic selectivity over the use of a control vector without miRNA regulation. Thus, incorporating miRNA regulation into a transcriptional targeting vector adds an extra layer of security to prevent off-target transgene expression and should be useful for the development of NSC vectors with high targeting specifcity for cancer therapy.

## 1. Introduction

Recent decade has witnessed the great progress of neural stem cell- (NSC-)based cancer gene therapy. NSCs are multipotent cells that give rise to the three principal neural lineages, neurons, astrocytes and oligodendrocytes, throughout the nervous system. When brain tumors are present, NSCs are capable of migrating through the brain parenchyma to home in on tumor foci by using cytokines, chemokines, and growth factors released from the tumor sites as candidate migration stimulatory signals. In animal tumor models, the innate tropic behavior of NSCs for tumors has been exploited extensively for targeted delivery of therapeutic genes not only to brain tumors, but also to peripheral nerve tumors and solid tumors of a nonneural origin. In these studies, intratumorally or intravenously injected NSC vectors expressing a therapeutic transgene, such as the suicide gene thymidine kinase (TK), are demonstrated to significantly suppress the growth of various solid malignant tumors via strong bystander effects [1–4]. However, further biodistribution experiments using mouse breast cancer models showed that the majority of intravenously injected NSCs became trapped in some nontarget organs, such as lung, liver, spleen, and kidney, probably due to the narrow diameters of capillaries in these organs [1, 5, 6]. Moreover, constant expression of the suicide gene TK from these off-target NSCs caused significant cytotoxic effects on liver and kidney [1]. Thus, it raises a safety concern that the use of these NSC vectors may deteriorate the situations of the patients. To harness the cytotoxic effect of these NSC vectors, an expression cassette that triggers tumor site-specific transgene expression is desired.

In view of the recent finding that hypoxia plays a key role in the directed migration of NSCs towards tumors [7], hypoxia-targeting approach can be employed to regulate transgene expression in NSC vectors. Hypoxia is a hallmark of the tumor microenvironment. In most human tissues, the physiological oxygen tensions range from 2% to 9%. However, due to the high metabolic rate, the oxygen tensions in tumor sites can fall far below the normal physiological levels, even down to 0.1% in necrotic regions [8]. Hypoxia correlates positively with tumor aggressiveness and has been implicated in inducing secretion of angiogenic factors, activating metabolic shift to anaerobic glycolysis, promoting epithelial-to-mesenchymal transition, remodeling extracellular matrix, and providing a selective survival advantage to cancer stem cells [9–11]. The dominant regulator of cellular response to hypoxia stress is hypoxia-inducible factors (HIFs), which bind to hypoxia-responsive elements (HREs) (5′-A/GCGTG-3′). HREs are minimal cis-regulatory elements that mediate transcriptional activation of more than 60 genes crucial to systemic hypoxia responses [12]. Some of these hypoxia-responsive genes, such as CXCR4 and VEGF, are closely correlated to the regulation of tumor tropism of NSCs [7]. Therefore, HREs can be used as a means for transcriptional targeting of tumor hypoxia.

MicroRNAs (miRNA) are alternative regulators of the cellular response to hypoxia stress. MiRNAs are endogenous small noncoding RNAs of 22~23 nucleotides that suppress mRNA translation by targeting complementary sequences in 3′-untranslated region (3′UTR). It has been described that hypoxia can cause miRNA expression alternations in cells. For instance, miRNA-210 is stimulated under hypoxic condition and regulates E2F3, an important protein in cell cycle, in breast and ovarian cancers [13, 14]. On the contrary, miRNA-199a-5p is reported to be acutely downregulated and derepress its target HIF1A in cardiac myocytes upon hypoxia treatment [15]. Recently, a series of studies have demonstrated that by incorporating properly engineered targeting sequences for a highly expressed endogenous miRNA into the 3′UTR of the expression cassette, the transgene expression can be suppressed up to 100-fold in a cell type-specific manner [16–18]. Thus, artificial miRNA targeting sequences have emerged as a powerful new tool for regulation of transgene expression.

To realize the full potential of NSC-based cancer therapy, it is desirable to develop approaches that enable precise control of transgene expression. We reason that the use of a hypoxia-responsive promoter could be a means to direct transgene expression preferentially to hypoxic tumor sites. Furthermore, the use of miRNA regulation, adjunct to the use of the hypoxia-responsive promoter, would provide the second layer of control to differentiate transgene expression between tumor sites and healthy organs. To test the hypothesis, we constructed an NSC vector platform that couples hypoxia-responsive promoter with miRNA regulation for tumor hypoxia-targeted gene therapy. The goal was to drive transgene expression in hypoxic tumor site, while sparing other healthy organs in case of local or systemic leakage of intratumorally injected vectors. We show that this combinatorial control using transcriptional targeting and miRNA regulation resulted in negligible off-target transgene expression in a mouse tumor model.

## 2. Materials and Methods

### 2.1. iPS Cell Generation and Neural Differentiation

An induced pluripotent stem (iPS) cell line was derived from the skin fibroblasts of a healthy person as described previously [19]. Briefly, skin fibroblasts were infected with viral supernatants generated by transfection of HEK293T cells using Lipofectamine 2000 (Invitrogen, Carlsbad, CA, USA) with retroviral pMXs vector (AddGene, Cambridge, MA, USA) containing the cDNAs of human OCT4, SOX2, KLF4, and c-MYC. Two rounds of infection were performed successively (12 h each). Polybrene (4 mg/mL, Sigma-Aldrich, St. Louis, MO, USA) was added to increase infection efficiency. After the second round of infection, cells were trypsinized and seeded onto a layer of mouse embryonic fibroblasts cell (MEF) feeders in a 10 cm culture dish using hES cell medium. The medium was changed every day. From day 12 to day 18, those colonies, which were large enough and identifiable as hES cell-like, were picked mechanically and expanded in hES medium on feeders. The iPS clones were characterized by alkaline phosphatase (AP) activity staining and immunostaining for SSEA-3, SSEA-4, TRA-1-60, and TRA-1-81. Then the iPS cells were routinely passed every 4-5 days, and the medium was changed every day.

NSCs were derived from human iPS cells using an adherent monoculture differentiation method as described previously [5]. In brief, iPS cell colonies were detached from the 6-well cell-culture plate 7 days after plating by mechanical cutting. Then, iPS cells were dissociated using TrypLE express dissociation enzyme (Invitrogen) and plated onto a 0.1% gelatin-coated 6-well cell-culture plate at a density of 10^6^ per well and cultured in NSC medium, which was a 1 : 1 mixture of DMEM/F12 (Invitrogen) supplemented with 2% B27 (Invitrogen), 2 mM L-glutamine, 50 U/mL penicillin, 50 *μ*g/mL streptomycin, 20 ng/mL EGF (Sigma-Aldrich), and 20 ng/mL bFGF (Invitrogen). Half of the cell-culture medium was changed every 2 days. After 7 days of differentiation, the cells reached 90% confluence and were split at ratio of 1 : 2. After 1 month of expansion, NSCs were derived from iPS cells. NSCs were digested using TrypLE for cell passage and subcultured at ratio of 1 : 2 twice weekly.

### 2.2. Cell Culture

MCF-7 breast cancer cell line was maintained in Dulbecco's modified Eagle's medium (DMEM) supplemented with 10% fetal bovine serum (FBS), 2 mM L-glutamine, 50 U/mL penicillin, and 50 *μ*g/mL streptomycin. For hypoxia treatment, cell cultures were incubated in a hypoxia chamber (STEMCELL Technologies) filled with an anaerobic gas mixture of 94% N_2_, 5% CO_2_, and 1% O_2_.

### 2.3. In Vitro Boyden Chamber Cell Migration Assay

In vitro migration assays used the MCF-7 breast cancer cell line as an attractant and was performed in Boyden chambers with the BD Falcon HTS FluoroBlok 24-well Multiwell Insert System with 8 mm pore size (BD Falcon). One day before the assays, MCF-7 cells were suspended in serum-free Opti-MEM (Invitrogen) and seeded into a 24-well companion plates at a cell density of 2.5 × 10^5^ per well. NSCs were labeled with Calcein-AM (Invitrogen). The labeled NSCs were suspended in serum-free Opti-MEM and seeded into the Boyden chamber Transwell inserts at a cell density of 5 × 10^4^ per insert. After 24 h of culture at 37°C in 5% CO_2_, the fluorescent cells on the bottom side of the inserts (corresponding to migrating cells) were counted and the migration rate was calculated. All experiments were conducted in 6 replicates.

### 2.4. Plasmid Constructs

pGL4.11 (Promega) carrying the luc2P reporter gene was used as a starting backbone to construct the combinatorial transgene expression cassettes through multistep subcloning. Firstly, all promoters used in this study were placed upstream of the reporter gene. Secondly, 4× miRNA targeting sequences (mirT) were designed to be perfectly complementary to the respective miRNA (in lowercase in Table 1) with 3 different linkers spacing each targeting sequence. The respective sense and antisense strands of the 4× mirT oligonucleotides were phosphorylated, annealed, and then inserted downstream of the reporter gene. A control scramble targeting sequence (ScrT) of the same size was designed based on the lack of significant similarity to any known miRNA and subcloned into the same region (Table 1).

### 2.5. Luciferase Assay

Subconfluent cells in 48-well plate were transfected with plasmids encoding luciferase reporter gene at 400 ng per well, using 1.2 *μ*L Fugene 6 (Roche) according to the manufacturer's protocol. After 24 h, the cells were lysed by freeze-thaw method and the supernatants were measured for luciferase activity using Luciferase Assay System (Promega) according to the manufacturer's instructions. All samples were assayed 3 times in triplicate.

### 2.6. qPCR

For mRNA qRCR, total RNA was extracted using TRIzol (Invitrogen) according to the manufacturer's protocol. First-strand cDNA was synthesized using the SuperScript III First-Strand Synthesis System for RT-PCR (Invitrogen). 1 *μ*L of cDNA reaction mix was subjected to PCR amplification using PCR SuperMix (Invitrogen). The forward and reverse primers for qPCR analysis were listed in Table 2. GAPDH was selected as the internal reference gene for PCR quantification.

For miRNA qPCR, small RNA was isolated using PureLink microRNA Isolation Kit (Invitrogen) and treated with TURBO DNA-free DNase (Ambion). PolyA tailing and cDNA synthesis of the DNase-treated small RNA were performed using Ncode VILO microRNA cDNA Synthesis Kit (Invitrogen) according to the manufacturer's protocol. The forward primers for qPCR analysis were designed based on entire known mature miRNA sequence, with additional 3 “A”s at the 3′ end to improve amplification specificity (Table 2). The reverse primer used was the universal primer in the EXPRESS SYBR GreenER microRNA qRT-PCR Kit (Invitrogen). 5S rRNA was selected as the internal reference gene for PCR quantification. To determine absolute copy number, a standard curve was generated using a synthetic LIN-4 RNA oligonucleotide.

qPCR was performed on iQ5 RT-PCR detection system (BioRad). All reactions were run in triplicate.

### 2.7. Animal Experiment

Three adult female BALB/c athymic, immunoincompetent nude mice (weight 20 g; aged 6–8 weeks) were used. To establish an orthotopic mouse model of breast tumor, 1 × 10^6^ MCF-7 cells were injected into the right mammary fat pad and sham injection was given on the contralateral side. After 1 week, the tumor developed and 1 × 10^6^ NSC vectors were injected into the tumor site and into sham injection site, respectively. After 24 h, in vivo transgene expression levels were measured by the Xenogen IVIS-100 bioimaging system (Caliper).

### 2.8. Statistical Analysis

All data are represented as mean ± s.d. The statistical significance of differences was determined by Student's *t*-test or the two-factor analysis of variance (ANOVA). A *P* value of <0.05 was considered to be statistically significant.

## 3. Results

### 3.1. Generation of iPS-Derived Tumor-Tropic NSCs

**Figure 1:**
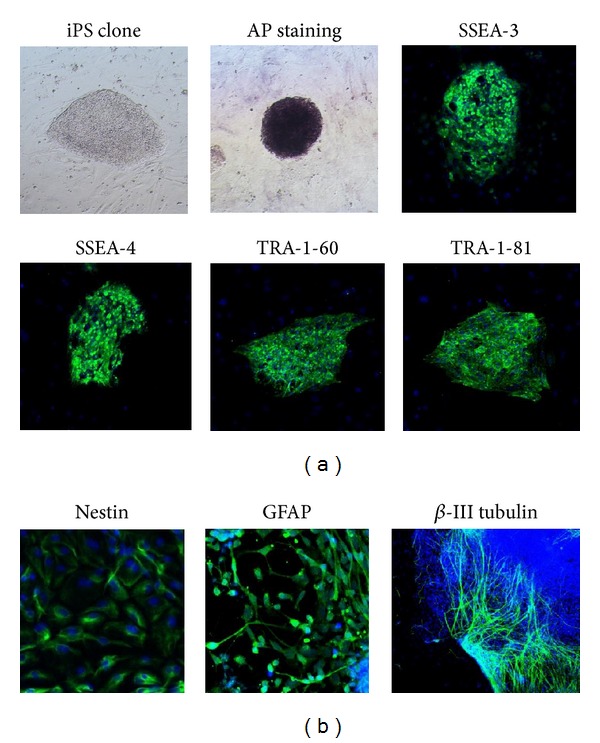
iPS cell line characterization and neural differentiation in vitro. (a) iPS cell line characterization. From left to right: iPS cell colony, AP staining, immunostaining to show protein expression of embryonic stem cell markers SSEA-3, SSEA-4, TRA-1-60, and TRA-1-81. (b) Neural differentiation in vitro. From left to right: immunostaining to show protein expression of NSC early stage marker nestin, NSC differentiation markers GFAP (glial cell marker), and *β*-III tubulin (neuron marker).

For the derivation of NSCs, an induced pluripotent stem (iPS) cell line was created from skin fibroblasts by retroviral transduction of the four Yamanaka's factors (Oct4, Sox2, Klf4, and c-Myc) [20] and characterized by AP staining and immunostaining for the pluripotency markers SSEA-3, SSEA-4, TRA-1-60, and TRA-1-81 (Figure 1(a)). Then NSCs were derived from the above iPS cells by a simple adherent monolayer culture method and characterized by immunostaining for nestin, an early-stage marker of NSCs (Figure 1(b)). After sequential withdrawal of bFGF and EGF, they generated a mixed population of glial fibrillary acidic protein- (GFAP-)positive glial cells and *β*-III tubulin-positive neurons (Figure 1(b)). Thus, the iPS-derived NSCs we obtained possessed the potential to differentiate into glial cells and neurons.

**Figure 2:**
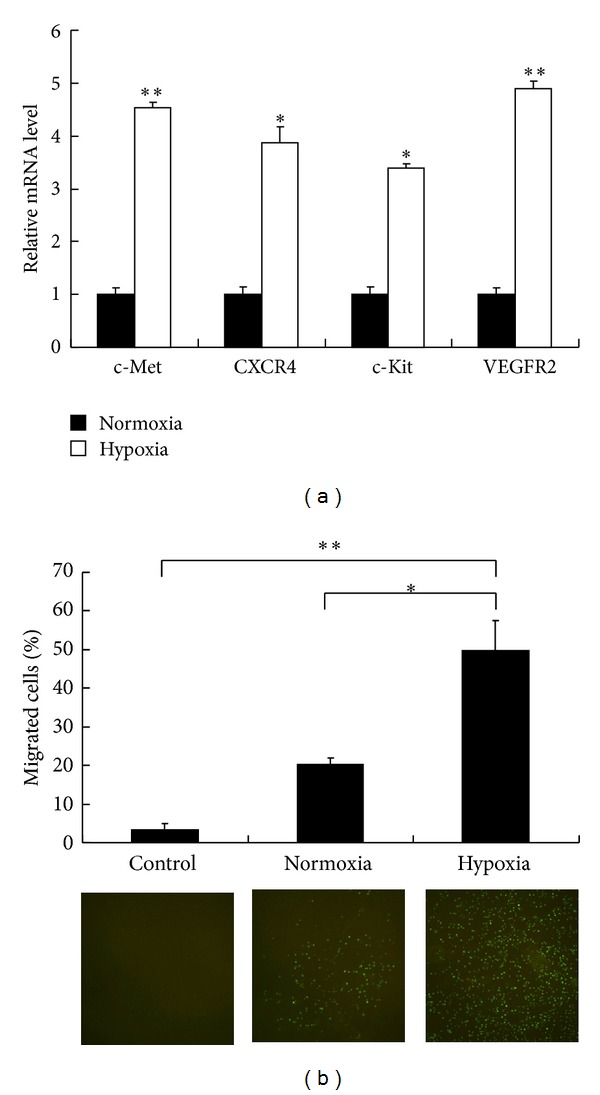
NSCs response to tumor hypoxia. (a) mRNA expression level of hypoxia-related signal receptors, c-Met, CXCR4, c-Kit, and VEGFR2, in NSCs cultured 24 h under normoxic and hypoxic conditions is quantified by qPCR. (b) Migration rates of NSCs towards MCF-7 breast cancer cells under normoxic and hypoxic conditions are quantified by Boyden chamber cell migration assays. MCF-7 cells were seeded in the lower chamber, and blank medium in the lower chamber was used for the negative control group. NSCs were stained with calcein-AM and seeded in the upper chamber. After 24 h, the labelled NSCs which migrated toward the lower chamber were evaluated (*n* = 5). Top: percentage of migrated NSCs. Bottom: fluorescence images showing the migration of NSCs toward the lower chambers. Error bars: s.d. *P < 0.05, **P < 0.01.

We then further characterized the tumor-tropic capacities of these iPS-derived NSCs under hypoxic condition. Since it is reported that the tumor tropism of NSCs is regulated by hypoxia-related signaling pathways [7], the mRNA expression alternation of hypoxia-related signal receptors, c-Met, CXCR4, c-Kit, and VEGFR2, in NSCs upon hypoxia treatment was measured by qPCR (Figure 2(a)). The results showed that the expression levels of all the above receptors were upregulated by 3-4 folds in NSCs after hypoxia treatment. Next, the in vitro migration ratio of NSCs towards MCF-7, a human breast cancer cell line, was measured by Boyden chamber cell migration assays (Figure 2(b)). The results displayed that under hypoxic condition, the percentage of migrated NSCs towards MCF-7 increased from 20% to 50%. Taken together, our results demonstrated that the iPS-derived NSCs maintain the tumor-tropic migratory capacity which is probably stimulated by the hypoxia-related cytokines released from tumor cells.

### 3.2. OptHRP Is the Optimal Hypoxia-Responsive Promoter in NSCs

To explore the optimal hypoxia-responsive promoter for transcriptional targeting in the NSC vectors, we chose three candidate HRE-containing promoters based on literatures: (1) a 300 bp CXCR4 promoter [21]; (2) a 2 kb artificial promoter (VEGF) composed of 5× VEGF HREs followed by a miniCMV promoter [22]; (3) a 64 bp artificial promoter (optHRP) composed of 4× optimized HREs followed by a TATA box [23]. We cloned these promoter constructs into the pGL4.11 luciferase reporter plasmids and then transfected them into NSCs to test their activities under normoxic and hypoxic conditions, respectively. Another one driven by EF1*α* promoter, a strong promoter widely used for constitutive transgene expression in most human cell types, was included as a positive control. The promoterless basic pGL4.11 plasmid was used as a reference for minimal transgene expression.

**Figure 3:**
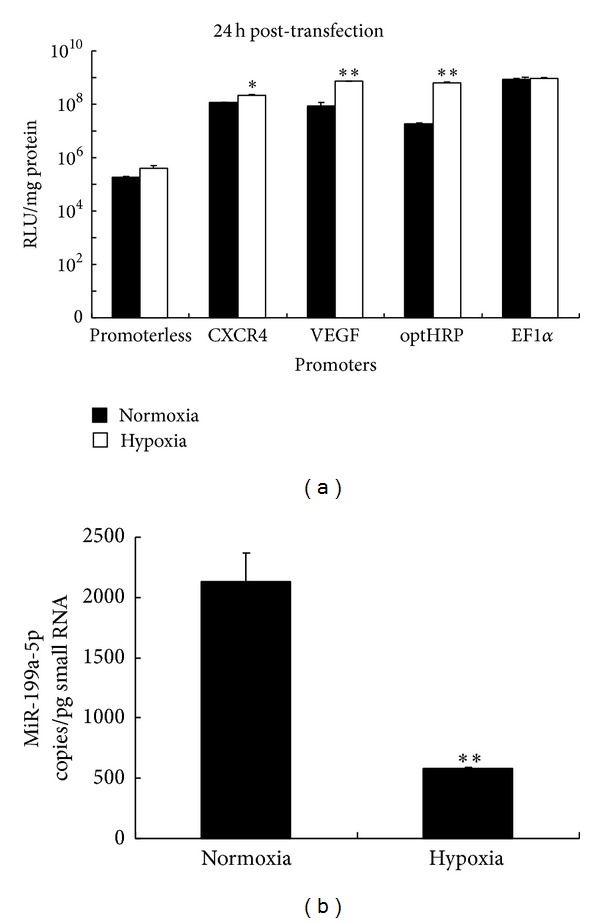
Selection of the optimal hypoxia-responsive promoter and normoxia-specific miRNA in NSCs. (a) Luciferase assays showing promoter activities. CXCR4 promoter, VEGF promoter, optHRP, and EF1*α* promoter were cloned into the pGL4.11 promoterless luciferase reporter plasmids, respectively. The promoterless pGL4.11 plasmid was included as negative control. NSCs were divided into 5 groups, transfected with the above constructs, and cultured 24 h under normoxic and hypoxic conditions, respectively. Then the promoter activities were quantified by luciferase assays. (b) Absolute expression levels of miR-199a-5p in NSCs under normoxic and hypoxic conditions are quantified by qPCR. miRNA copy numbers were calculated based on a standard curve generated using a synthetic LIN-4 RNA oligonucleotide. Abbreviation: RLU, relative luminescence unit. Error bars: s.d. *P < 0.05, **P < 0.01.

The luciferase assay results (Figure 3(a)) showed that all the three hypoxia-responsive promoters displayed lower activities under normoxic condition and could be induced under hypoxia treatment. Moreover, their hypoxia-induced activities were comparable to the EF1*α* promoter, which indicated that their activities were strong enough to drive robust transgene expression for therapeutic purpose. Among these promoters, optHRP exhibited the highest differential expression levels between normoxic and hypoxic conditions, up to 34 folds. Therefore, optHRP was selected as the optimal hypoxia-responsive promoter for NSC vectors. However, its basal activity in NSC vectors under normoxic condition was still 100 folds higher than that of the promoterless reference, which implied a promoter leakage at off-target sites. Since prolonged, though low, off-target transgene expression can result in cytotoxicity to surrounding healthy tissues, it is necessary to add a second layer of control to shut down the leaky transgene expression.

### 3.3. miR-199a-5p Is Enriched in NSCs and Downregulated upon Hypoxia Induction

In order to solve the problem of promoter leakage, we next screened for endogenous miRNAs that selectively express under normoxic condition to block off-target transgene expression. According to literature, miR-199a-5p is an inhibitor of HIF-1*α* and decreases in response to hypoxia in cardiac myocytes [15]. Hence, we investigated whether it would be the same case in NSCs. Small RNA samples were extracted from NSCs under normoxic and hypoxic conditions, respectively. And the absolute copy number per pg small RNA of miR-199a-5p was quantified using qPCR. The results (Figure 3(b)) showed that miR-199a-5p was highly expressed in NSCs under normoxic condition, up to 2130 copies per pg small RNA. Based on previous report, an expression level above 100 copies per pg small RNA is sufficient for the endogenous miRNA to yield suppression effect on exogenous transgene expression [17]. In addition, the miR-199a-5p expression level in NSCs was dramatically downregulated by about 73% upon hypoxia treatment. Therefore, miR-199a-5p holds the potential to block transgene expression in a normoxia-specific manner.

### 3.4. Combinatorial Regulation of Transgene Expression In Vitro

**Figure 4:**
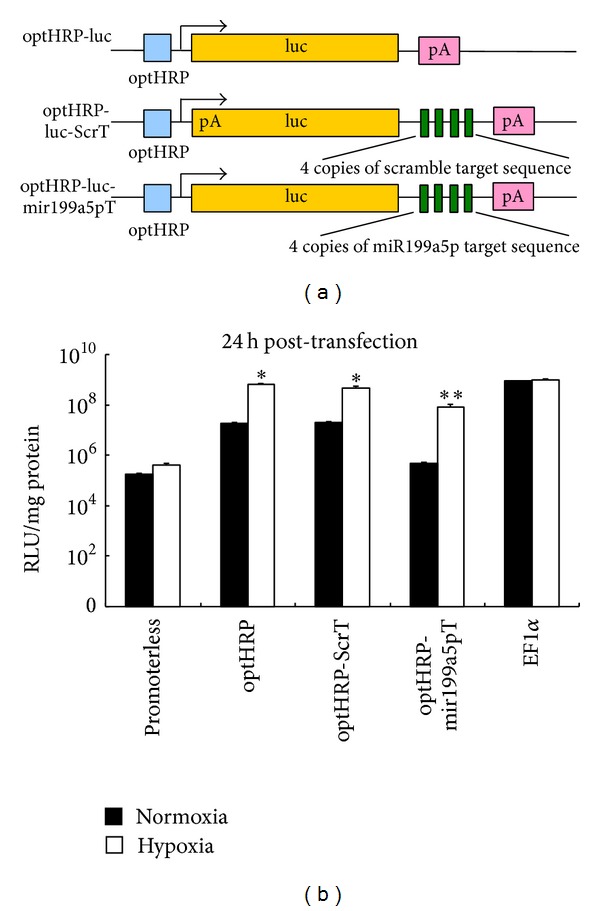
Combinatorial effect of optHRP and miR-199a-5p on transgene regulation in NSC. (a) Schematic representation of the combinatorial expression cassettes containing the optHRP promoter and miRNA target sequences. optHRP, an artificially optimized hypoxia-responsive promoter; luc, luciferase reporter gene; miRNA target sequences as detailed in Table 1 were inserted into the 3′UTR; pA, polyA signal. (b) Transgene expression levels of different expression cassettes within NSCs under normoxic and hypoxic conditions are quantified by luciferase assays. Abbreviation: RLU, relative luminescence unit. Error bars: s.d. *P < 0.05, **P < 0.01.

To employ both hypoxia-responsive promoter and miRNA regulation to restrict transgene expression to hypoxic condition, we constructed a combinatorial expression cassette (Figure 4(a)). We used the optHRP-luc plasmid generated in the previous promoter activity experiment and introduced perfectly complementary miRNA target sequences of miR-199a-5p into the 3′UTR of the luciferase reporter gene (optHRP-luc-mir199a5pT). To rule out the possibility of less favorable transcription caused by introduction of a long repeat sequence into the 3′UTR, we constructed a control plasmid optHRP-luc-ScrT by replacing the miR-199a-5p target sequences with a scramble target sequence of the same length, which was designed based on the lack of significant similarity to any known miRNA (Table 1).

After including the above regulatory elements into the luciferase reporter plasmids, we tested the ability of miRNA target sequences to repress luciferase gene expression in NSC vectors before and 24 h after hypoxia treatment. The promoterless reference plasmid and the EF1*α* positive control plasmid were included as previously mentioned. The results (Figure 4(b)) showed that the optHRP-miRNA199a5pT expression cassette obtained significantly higher hypoxic selectivity than that of the optHRP-luc expression cassette, from 34 folds up to 176 folds. Remarkably, its expression level at normoxic condition is suppressed to be as low as that of the promoterless reference plasmid. As a comparison, the insertion of scramble targeting sequence did not repress transgene expression under normoxic condition significantly. Taken together, our findings suggest that the optHRP-luc-mir199a5pT expression cassette is a hypoxia-inducible, nonleaky transgene expression cassette for NSC vectors. And the inhibition of transgene expression under normoxic condition is probably due to the binding of the artificially introduced target sequences to corresponding endogenous miRNAs but not due to transcriptional suppression.

### 3.5. Tumor Site-Specific Transgene Expression In Vivo

**Figure 5:**
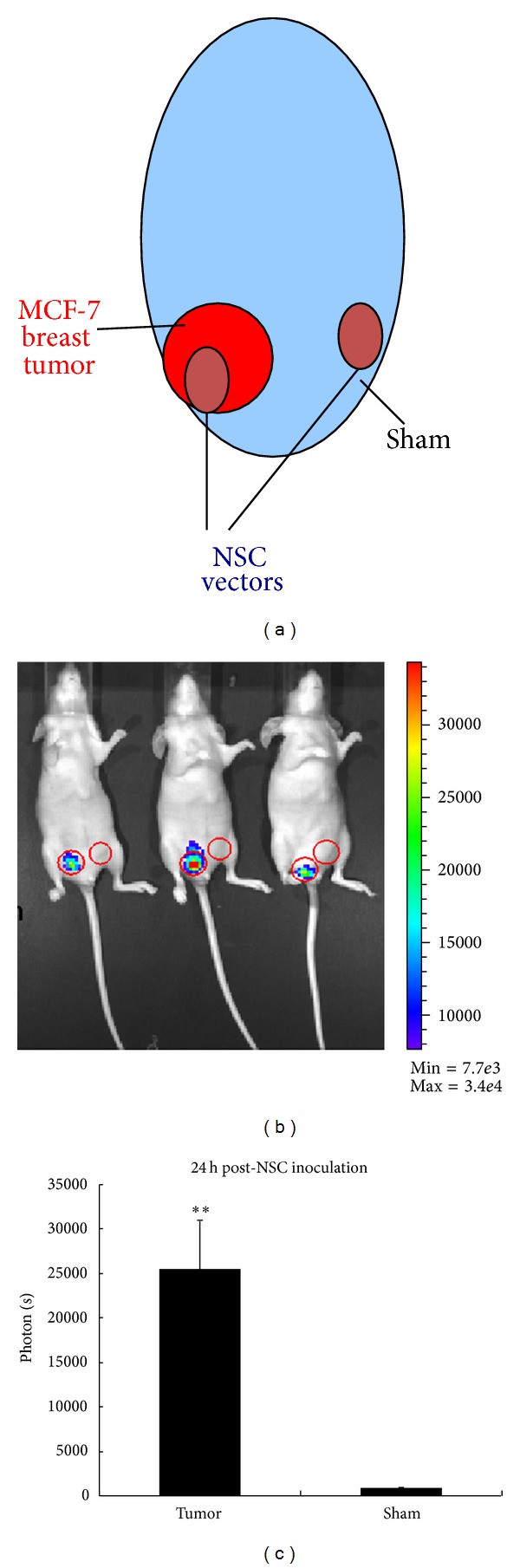
In vivo transgene expression in MCF-7 tumor-bearing mice. (a) Schematic representation of the in vivo expression assay. MCF-7 cells are inoculated into the right mammary fat pad of the mouse and sham injections are given on the contralateral side. After the tumors develop, NSC vectors are inoculated into the tumor sites and sham injection sites, respectively. 24 h after NSC inoculation, luciferase reporter gene expression levels are monitored by live animal imaging. (b) Bioluminescent image showing luciferase reporter gene expression in the tumor-bearing mice. Red circles indicate the inoculation sites of NSC vectors. (c) Average quantitative transgene expression levels in the tumor sites and sham injection sites. Error bars: s.d. *P < 0.05, **P < 0.01.

We next investigated whether the combinatorial NSC vectors can mediate tumor site-specific transgene expression in vivo. An orthotopic mouse model of breast cancer was established by inoculating MCF-7 breast cancer cells into the right mammary fat pad and sham into the left (Figure 5(a)). After the tumor developed, NSCs transfected with the optHRP-luc-mir199a5pT plasmid were injected into the tumor sites and sham sites, respectively. The next day, luciferase reporter gene expression levels were monitored by a live animal bioimaging system (Figure 5(b)). Quantitative results from all the three mice were summarized in Figure 5(c), showing that NSC vectors displayed induced luciferase gene expressions in tumor sites by averagely 30 folds higher than those in sham sites. Thus, the combinatorial NSC vectors are demonstrated to be capable of mediating tumor site-specific transgene expression in vivo.

## 4. Discussion

In this study, we report the successful construction of a novel expression cassette which triggers transgene expression within NSC vectors in a hypoxia-inducible manner. To the best of our knowledge, this is the first combinatorial transgene expression cassette designed for NSC vectors.

The great potential of NSCs in cancer therapy underlines the need for reliable and stable sources for the large scale production of human NSCs suitable for clinical applications. The use of human iPS cells has provided a robust and accessible source to produce unlimited amounts of human NSCs for cancer gene therapy [24]. iPS cell-based approach also helps bypass the sensitive ethical issue surrounding the use of human embryonic stem (hES) cells and the safety concern of immune rejection by allogeneic transplantation. In this study, an iPS cell line derived from skin fibroblasts was created and characterized for derivation of NSCs. The iPS-derived NSCs possessed the potential to differentiate into multiple neural lineages and could be genetically manipulated for the test of our system. More importantly, our in vitro Boyden chamber cell migration assays demonstrated that these NSCs maintained the tumor-tropic capacity as previously described, which indicated the feasibility of translating the iPS cell techniques into personalized therapies. Intriguingly, our NSCs displayed a significantly higher migratory rate towards tumor cells under hypoxic condition. Based on the recent findings that hypoxia plays an essential role in the maintenance and growth of cancer stem cells [10, 25–27], there has been an emergent hypothesis that cancer stem cells are concentrated in the more alkaline hypoxic regions of a tumor mass [28]. Therefore, the use of hypoxia-inducible transgene expression cassette in NSC vectors may offer an additional advantage in targeting cancer stem cells.

Previous studies have extensively exploited hypoxia-responsive promoters for transcriptional targeting of tumor hypoxia. In this study, we tested the CXCR4 promoter [21], an engineered VEGF promoter [22] and an artificially optimized promoter optHRP [23] based on literatures. Although optHRP displayed the highest hypoxic selectivity (34 folds) among these promoters in our NSCs, its basal activity under normoxic condition was still detectable, approximately 100 folds higher than the promoterless reference. This finding indicates that even after careful design and artificial engineering, “promoter leakage” is still hard to avoid. Since NSC vectors trapped in off-target organs may survive for up to 14 days even in immunocompetent mice [5], the prolonged, though low, off-target transgene expression may cause decent damages in the these organs. Hence, a second layer of transgene control on top of transcriptional targeting is necessary.

Substantial studies from different groups have demonstrated the feasibility of employing endogenous miRNA to inhibit transgene expression in a cell type-specific manner [16–18, 29]. However, our study is the first to demonstrate that endogenous miRNA can also be exploited to block transgene expression in a condition-specific manner, as miRNA expression level alters in response to cell stress. In this study, we showed that incorporation of miR-199a-5p targeting sequences into hypoxia-inducible expression cassette successfully enhanced the specificity from 34 folds to 176 folds in NSCs. Remarkably, the transgene expression level under normoxic condition was suppressed to a minimal level as low as the promoterless reference, which implied the effectiveness of using endogenous miRNA regulation to diminish the off-target transgene expression. Therefore, this combinatorially regulated transgene expression cassette represents a valuable tool for the NSC-mediated gene delivery, as it provides a feasible solution for the safety concern on the off-target transgene expression and makes the NSC vectors more translatable for clinical application.

Besides the transcriptional and post-transcriptional regulations, other additional approaches can also be employed to enhance the hypoxic selectivity of the transgene expression cassette. For example, the oxygen-dependent degradation (ODD) domain can be employed as a post-translational regulator to suppress transgene expression under normoxic condition [30]. The ODD domain derived from HIF1A gene can be inserted downstream of the transgene and facilitate degradation of the fusion protein product in cells under physiological oxygen concentration, thus adding an extra barrier on off-target transgene expression.

## 5. Conclusion

In summary, this report demonstrates an inducible, nonleaky expression cassette which functions within NSC-based gene delivery vectors for tumor site-specific transgene expression. Our data exhibit that it is able to selectively trigger reporter gene expression under hypoxic condition in vitro and at tumor sites in vivo. Most importantly, a negligible transgene expression level in nontarget region is observed, indicating the high safety of applying this regulatory system for NSC-mediated gene delivery. Further refinement of this system may lead to the development of optimal cell-based gene delivery vector to target malignant tumors.

## Figures and Tables

**Table 1:** MicroRNA targeting (mirT) sequences.

mirT		Sequence
miR-199a-5p	S1	5′-CTAGATAAgaacaggtagtctgaacactgggCGATgaacaggtagtctgaacactggg-3′
	S2	5′-ACGCGTgaacaggtagtctgaacactgggTCACgaacaggtagtctgaacactgggGCATG-3′
	AS1	5′-ACGCGTcccagtgttcagactacctgttcATCGcccagtgttcagactacctgttcTTAT-3′
	AS2	5′-CcccagtgttcagactacctgttcGTGAcccagtgttcagactacctgttc-3′

ScrT	S1	5′-CTAGAtaatttatgatctgcgcgtggagacgcccgattttatgatctgcgcgtggagacgcc-3′
	S2	5′-acgcgttttatgatctgcgcgtggagacgcctcactttatgatctgcgcgtggagacgccGCATG-3′
	AS1	5′-acgcgtggcgtctccacgcgcagatcataaaatcgggcgtctccacgcgcagatcataaattaT-3′
	AS2	5′-Cggcgtctccacgcgcagatcataaagtgaggcgtctccacgcgcagatcataaa-3′

**Table 2:** qPCR primers.

Gene symbol	Primer sequence
MET	F: 5′-TGATGATGAGGTGGACACA-3′
	R: 5′-ATTTTGGCAAGAGCAAAGA-3′
CXCR4	F: 5′-CAAGGCCCTCAAGACCACA-3′
	R: 5′-CCCAATGTAGTAAGGCAGCCAA-3′
KIT	F: 5′-CGCCTGGGATTTTCTCTGC-3′
	R: 5′-TCACAGATGGTTGAGAAGAGCCT-3′
VEGFR2	F: 5′-CGGCTCTTTCGCTTACTGTTCT-3′
	R: 5′-AGCATGGAAGAGGATTCTGGACT-3′
miR-199a-5p	F: 5′-CCCAGTGTTCAGACTACCTGTTCAAA-3′

F: forward primer; R: reverse primer.
